# Patterns and predictors of end‐of‐life care in older patients with pancreatic cancer

**DOI:** 10.1002/cam4.1861

**Published:** 2018-11-13

**Authors:** Ryan D. Nipp, Angela C. Tramontano, Chung Yin Kong, Chin Hur

**Affiliations:** ^1^ Division of Hematology and Oncology, Department of Medicine Massachusetts General Hospital Cancer Center Boston Massachusetts; ^2^ Harvard Medical School Boston Massachusetts; ^3^ Institute for Technology Assessment Massachusetts General Hospital Boston Massachusetts

**Keywords:** end‐of‐life, hospice, pancreatic cancer, SEER‐Medicare, treatment

## Abstract

**Background:**

Little is known about end‐of‐life care among patients with pancreatic adenocarcinoma (PDAC). We used the Surveillance, Epidemiology, and End Results‐Medicare linked database to analyze patterns of hospice use and end‐of‐life treatment in patients with PDAC.

**Methods:**

We included patients diagnosed with PDAC between 2000‐2011 and who had died by December 31, 2012. We assessed patterns of hospice use, chemotherapy receipt, and intensive care unit (ICU) admissions at end‐of‐life. We used multivariable logistic regression to investigate predictors of end‐of‐life care.

**Results:**

In our cohort of 16 309 patients, 70.5% enrolled in hospice, of which 29.1% enrolled in the last 7 days of life. Use of hospice increased over time, from 61.6% in 2000 to 77.5% in 2012 (*P*‐value for trend <0.0001). Among the entire cohort, 6.4% received chemotherapy within the last 14 days of life and 13.1% were admitted to the ICU within the last 30 days of life. Late ICU admissions increased over time, while chemotherapy receipt at the end‐of‐life decreased. Patients who were older, female, with higher SES, or from the South or Midwest were more likely to enroll in hospice. Those who were younger or male were more likely to receive chemotherapy or have an ICU admission at the end‐of‐life.

**Conclusion:**

Although hospice enrollment has increased among patients with PDAC, late enrollment still occurs in a substantial proportion of patients. While chemotherapy at the end‐of‐life has decreased slightly, ICU admissions at the end‐of‐life have continued to increase. Further research is needed to determine effective ways of enhancing end‐of‐life care for patients with PDAC.

## 1 INTRODUCTION

Pancreatic ductal adenocarcinoma (PDAC) is a remarkably aggressive and fatal disease. More than two‐thirds of patients with PDAC have incurable disease at diagnosis, and historically over 90% of patients die within 5 years.^1^, ^2^, ^3^ PDAC is the third leading cause of cancer death in the United States and will likely become the second leading cause of cancer death by 2030.^4^ In 2017, an estimated 53 670 new PDAC diagnoses are expected in the United States, and 43 090 deaths will be attributed to PDAC.^5^ Notably, incidence rates have been increasing, with a rate of 1.2% per year between 2000‐2012, and concurrently death rates have increased by 0.4%.^1^ Thus, ongoing research is critically needed to help alleviate suffering and improve outcomes for these patients.

Surgical resection remains the only curative treatment option for patients with PDAC; however, only approximately 20% present with resectable disease.^6^, ^7^, ^8^ Tumors located in the tail and body historically have been associated with a poorer prognosis than those located in the pancreas head, theoretically due to their later clinical presentation and lower rates of resectability.^9^, ^10^ For patients with incurable PDAC, chemotherapy is the primary treatment option. In the past, single chemotherapeutic agents, such as gemcitabine, were the standard of care for patients with incurable PDAC. In recent years, trials of newer chemotherapeutic agents in combination have demonstrated improved outcomes for patients with PDAC, including FOLFIRINOX. However, PDAC remains difficult to treat and survival rates remain low.

Effective end‐of‐life care is essential for patients with cancer, and discussions surrounding high‐quality end‐of‐life care include considerations about appropriate use of treatment and timely use of hospice services (ideally prior to the final week of life).^11^, ^12^, ^13^, ^14^, ^15^ Optimizing end‐of‐life care is particularly important for patients with PDAC, as they often experience substantial symptom burden including pain, fatigue, poor appetite, and nausea.^16^, ^17^, ^18^ Research has demonstrated that during the final months of life, patients with PDAC can experience increased symptom burden and decreased overall quality of life.^19^ Hospice services can help manage symptom burden and enhance the quality of life for patients at the end of their life.^20^, ^21^, ^22^ However, a large proportion of patients with cancer do not receive hospice care, or enroll late, despite increasing awareness of the importance of hospice services.^23^, ^24^, ^25^ Therefore, patients miss the opportunity to receive all the benefits and support that hospice provides. In contrast, evidence suggests that aggressive end‐of‐life care has increased in recent years for patients with cancer, including chemotherapy receipt, hospitalizations, and intensive care unit (ICU) admissions, potentially related to the introduction of newer treatment options.^26^, ^27^, ^28^, ^29^, ^30^, ^31^

We used the Surveillance, Epidemiology, and End Results (SEER)‐Medicare linked database to investigate end‐of‐life care among patients with PDAC. Specifically, we sought to evaluate the timing of hospice enrollment, receipt of anticancer treatment at the end‐of‐life (chemotherapy and radiation within the last 14 days of life), and rates of hospitalizations and ICU admissions within the last 30 days of life.^14^, ^32^ We explored whether certain sociodemographic and clinical characteristics are associated with patients’ end‐of‐life outcomes. We hypothesized that hospice use may be rising in recent years, given increased recognition of the importance of hospice care, yet aggressive end‐of‐life care (eg, anticancer treatment and health care utilization in the final days of life) would be increasing as well.

## 2 METHODS

### 2.1 Cohort inclusion/exclusion

This study was approved by the Institutional Review Board at Massachusetts General Hospital.

A description of the SEER‐Medicare database and an inclusion/exclusion flowchart can be found in the Appendix S1. We included patients with PDAC (a) with International Classification of Diseases for Oncology (ICD‐O‐3) adenocarcinoma histology codes (table 1 in Appendix S2) and pancreatic cancer as the primary cancer, (b) diagnosed at 66 or older between 2000‐2011 and who died by December 31, 2012, and (c) had continuous enrollment in Medicare Parts A and B for 13 months prior to diagnosis to death, and without HMO during this period. Those diagnosed at autopsy or death were excluded. We determined cancer stage using the SEER stage variable for the sixth edition of the AJCC Cancer Staging Manual. Those diagnosed prior to 2004 were mapped to the appropriate AJCC 6th edition stage using variables for extension of disease and lymph node involvement. We excluded unknown cancer stage.

### 2.2 Statistical analysis

We conducted all statistical analyses using SAS version 9.4 (SAS Institute, Inc, Cary, NC). We considered patient and clinical characteristics that may predict hospice enrollment and end‐of‐life care receipt: age, sex, race/ethnicity, marital status, SEER region, urban location, ecological socioeconomic status (SES), AJCC stage, comorbidity score, and tumor location. We calculated comorbidity scores using the Deyo adaptation of the Charlson comorbidity index.^33^, ^34^, ^35^ We used US Census data provided in SEER‐Medicare to derive quintiles of ZIP code‐level median household income to impute ecological SES as a proxy for patient SES.

We investigated aggressive end‐of‐life treatment receipt using indicators established as measures of end‐of‐life care by the National Quality Forum, which have been used in earlier studies: chemotherapy receipt within the final 14 days, radiation receipt within the last 14 days of life, any acute care hospitalizations within the last 30 days of life, at least two acute care hospitalizations within their final 30 days, and an ICU admission within the final 30 days of life.^11^, ^30^, ^32^, ^36^, ^37^, ^38^ We defined hospice enrollment, hospitalizations, ICU admissions, chemotherapy, and radiation based on definitions in table 1 in Appendix S2. We defined late hospice enrollment as enrollment occurring within the last 7 days of death, and also investigated enrollment within 3 days of death.^14^, ^30^

We compared distributions of patient and clinical characteristics among patients with and without hospice enrollment using chi‐square tests. We evaluated hospice enrollment and end‐of‐life treatment receipt using the Cochran‐Armitage test to analyze trends over time. We used multivariable logistic regression models to identify predictors of hospice enrollment in the entire cohort and late hospice enrollment among those enrolled, and to evaluate associations between patient sociodemographic and clinical characteristics and aggressive end‐of‐life treatment (chemotherapy receipt within the last 14 days of life, and hospitalizations and ICU admission within patients’ last 30 days of life). To account for multiple testing, we used a Bonferroni correction with each of the models, using a *P*‐value of 0.01 (0.05/5) to test their significance.

## 3 RESULTS

### 3.1 Cohort characteristics

Our cohort included 16 309 patients with PDAC. The majority were White (83.2%). Over half were age 75 and above, female, married, lived in a large metropolitan area or had stage IV disease (Table 1). Among the entire cohort, 22.7% received radiation and 53.8% received chemotherapy at any time during their cancer course. Among all patients, 79.7% had an acute care hospitalization and 49.0% had an ICU admission at any time following diagnosis.

**Table 1:** Distribution of patient characteristics

Characteristic	Any hospice (N = 11 460) (%)	No hospice (N = 4 849) (%)	*P*‐value
Age (at death)			
66‐69	13.7	16.7	<0.0001
70‐74	25.2	28.1	
75‐79	26.8	26.4	
80‐84	21.3	18.9	
85+	13.1	9.9	
Sex			
Male	42.3	50	<0.0001
Female	57.7	50	
Race/ethnicity			
White	85.1	78.8	<0.0001
Black	8.4	10.9	
Hispanic	2.0	2.5	
Asian/other[Link]	4.5	7.9	
Marital status			
Unmarried	42.7	39.6	0.01
Married	55.0	57.5	
Unknown	3.3	2.9	
AJCC stage			
I	6.9	6.8	0.002
II	29.8	32.7	
III	8.8	7.8	
IV	54.5	52.8	
Charlson score			
0	39.5	36.1	<0.0001
1	31.2	29.5	
2+	29.3	34.4	
Year of death			
2000‐2004	30.6	37.2	<0.0001
2005‐2008	35.3	34.4	
2009‐2012	34.1	28.3	
SEER region			
Northeast	22.7	25.4	<0.0001
South	23.9	19.5	
Midwest	15.1	10.2	
West/Hawaii	38.3	45.0	
Residence			
Large metropolitan	55.5	59.3	<0.0001
Metro/urban	34.9	32.2	
Less urban/rural	9.6	8.5	
SES (Census tract quintile)			
0 (lowest)	19.0	23.2	<0.0001
1	19.0	20.6	
2	20.2	19.2	
3	20.7	18.7	
4 (highest)	21.0	18.3	
Pancreas location			
Head	51.0	53.2	0.007
Tail/body	25.3	23	
Other	23.7	23.8	

Includes 81 patients of other/unknown race/ethnicity.

### 3.2 Hospice enrollment

**Figure 1:**
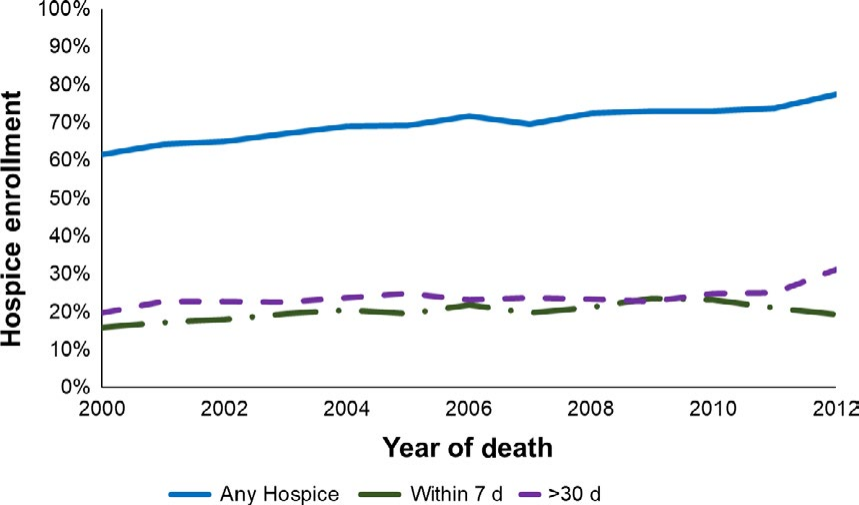
Hospice care among patients with PDAC from 2000 to 2012

Overall, 70.3% received hospice services prior to death. However, among enrollees, we found high rates of late enrollment, with 15.4% receiving hospice services within 3 days of death and 29.1% receiving hospice services within 7 days of death. The percentage of hospice‐enrolled patients increased from 61.6% in 2000 to 77.5% in 2012 (*P*‐value for trend <0.0001) (Figure 1). Patients enrolling >30 days before death increased, from 19.7% in 2000 to 31.2% in 2012 (*P*‐value for trend <0.0001). Those enrolling within 7 days of death increased, from 15.9% in 2000 to 19.3% in 2012, with a peak of 23.5% in 2009 (*P*‐value for trend <0.0001).

In multivariable logistic regression models exploring hospice enrollment, patients who were older, female, died in later years, lived in the South or Midwest (vs Northeast), lived in a Metro/Urban area (vs large metro), had higher SES (vs lowest), had a tail/body tumor (vs head), or had longer survival were more likely to enroll (Table 2), while Black and Asian patients were less likely to enroll (vs Whites) and those with a 2 + Charlson score were less likely to enroll (vs score 0). In multivariable logistic regression models comparing late hospice enrollment among enrollees, patients who were older, female, lived in the South or West/Hawaii (vs Northeast), lived in a Metro/Urban or Less Urban/rural area (vs large metro), or survived longer were less likely to have a late enrollment, while patients who were married, had stage IV disease (vs I), or died in later years were more likely to have a late enrollment. Similar results were seen when the time between hospice enrollment and death was restricted to 3 days (table 2 in Appendix S2), with a few exceptions. Namely, the South, Stage IV, Metro/Urban, and later years of death were not significantly associated with hospice enrollment within 3 days of death; patient with a 2 + Charlson score were less likely to enroll within 3 days (vs score 0).

**Table 2:** Characteristics associated with hospice enrollment, 2000‐2012[Link]

Characteristic	Hospice enrollment (N = 11 460)[Link]		Late hospice enrollment (N = 3 334)^b^	
	OR (95% CI)	*P*‐value	OR (95% CI)	*P*‐value
Age at death (ref = 66‐69)				
70‐74	1.10 (0.99‐1.23)	0.08	0.88 (0.77‐0.999)	0.048
75‐79	1.25 (1.12‐1.39)	0.0001	0.83 (0.73‐0.95)	0.007
80‐84	1.36 (1.21‐1.53)	<0.0001	0.68 (0.59‐0.79)	<0.0001
85+	1.58 (1.38‐1.82)	<0.0001	0.55 (0.46‐0.65)	<0.0001
Sex (ref = male)				
Female	1.34 (1.25‐1.44)	<0.0001	0.77 (0.71‐0.84)	<0.0001
Race/ethnicity (ref = white)				
Black	0.70 (0.62‐0.79)	<0.0001	1.10 (0.94‐1.28)	0.26
Hispanic	0.89 (0.70‐1.11)	0.30	0.75 (0.54‐1.03)	0.08
Asian/other	0.61 (0.53‐0.70)	<0.0001	0.99 (0.81‐1.21)	0.90
Marital status (ref = unmarried)				
Married	0.99 (0.92‐1.07)	0.75	1.14 (1.04‐1.25)	0.01
Unknown	1.08 (0.88‐1.32)	0.49	1.11 (0.88‐1.41)	0.38
AJCC stage (ref = I)				
II	0.97 (0.84‐1.12)	0.63	1.02 (0.85‐1.23)	0.80
III	1.18 (0.99‐1.41)	0.07	0.91 (0.73‐1.13)	0.40
IV	1.15 (0.996‐1.33)	0.06	1.26 (1.05‐1.51)	0.01
Charlson score (ref = 0)				
1	0.98 (0.90‐1.07)	0.68	1.07 (0.97‐1.18)	0.17
2+	0.80 (0.74‐0.87)	<0.0001	1.09 (0.99‐1.21)	0.08
Year of death (ref = 2000‐2004)				
2005‐2008	1.24 (1.14‐1.35)	<0.0001	1.10 (0.99‐1.22)	0.07
2009‐2012	1.46 (1.34‐1.60)	<0.0001	1.20 (1.08‐1.34)	0.0005
SEER region (ref = northeast)				
South	1.48 (1.32‐1.65)	<0.0001	0.67 (0.59‐0.76)	<0.0001
Midwest	1.78 (1.57‐2.02)	<0.0001	0.87 (0.75‐0.99)	0.04
West/Hawaii	1.01 (0.92‐1.10)	0.84	0.78 (0.70‐0.87)	<0.0001
Residence (ref = large metro)				
Metro/urban	1.15 (1.06‐1.24)	0.0005	0.89 (0.81‐0.98)	0.01
Less urban/rural	1.01 (0.95‐1.17)	0.87	0.69 (0.58‐0.81)	<0.0001
SES[Link] (ref = 0)				
1	1.06 (0.95‐1.17)	0.34	1.06 (0.92‐1.21)	0.42
2	1.19 (1.07‐1.33)	0.002	1.09 (0.95‐1.25)	0.20
3	1.21 (1.08‐1.36)	0.0008	1.03 (0.90‐1.19)	0.66
4 (highest)	1.27 (1.13‐1.42)	<0.0001	1.11 (0.97‐1.27)	0.15
Pancreas location (ref = head)				
Tail/body	1.11 (1.02‐1.22)	0.02	0.97 (0.88‐1.08)	0.61
Other	1.02 (0.93‐1.12)	0.64	1.03 (0.93‐1.15)	0.56
Survival (mo)	1.004 (1.00‐1.01)	0.03	0.99 (0.98‐0.99)	<0.0001

Models significant after Bonferroni correction.

AUC: 0.61 (both models).

Census tract quintile.

### 3.3 Aggressive end‐of‐life treatment

We investigated indicators of aggressive end‐of‐life care: chemotherapy or radiation receipt within 14 days of death and health care utilization within 30 days of death (ie, any acute care hospitalization, at least two acute care hospitalizations, or at least one ICU admission). We found that 6.4% of patients received chemotherapy and 1.5% of patients received radiation at the end‐of‐life; 45.9% had a late hospitalization, 10.1% had at least two late hospitalizations, and 13.1% had an ICU admission at the end‐of‐life.

**Figure 2:**
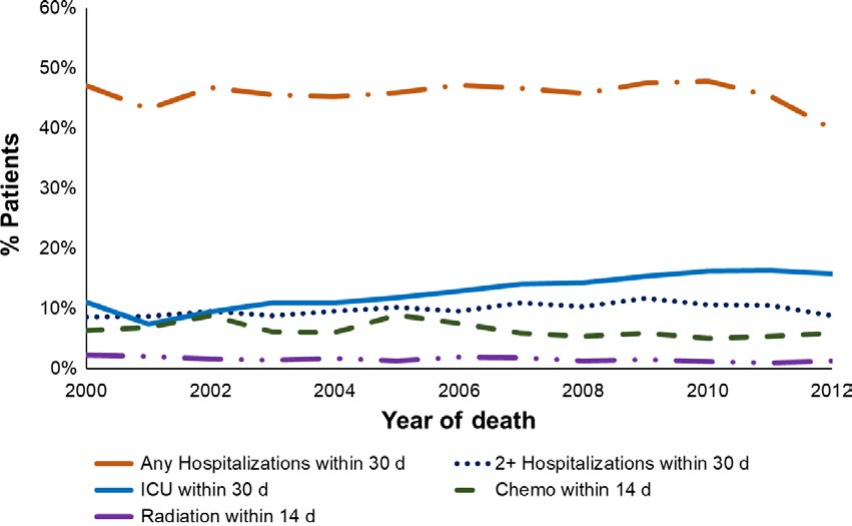
End‐of‐life treatment among patients with PDAC from 2000 to 2012

The percentage of patients receiving chemotherapy within 14 days of death increased from 6.4% in 2000 to 8.6% in 2005, before decreasing to 5.9% in 2012 (*P*‐value for trend <0.0001). Rates of late radiation receipt were low, decreasing from 2.3% in 2000 to 1.3% in 2012 (*P*‐value for trend = 0.01). Hospitalization rates within 30 days of death remained steady over time (*P*‐value for trend = 0.88), although we observed a slight drop in 2012. However, rates of two or more hospitalizations increased over time, from 8.7% to 8.9%, with a maximum rate of 11.9% in 2009 (*P*‐value for trend = 0.02). We also found an increase in ICU admissions within 30 days of death, from 11.1% in 2000 to 15.8% in 2012 (*P*‐value for trend <0.0001). All trends are displayed in Figure 2.

We found that patients who enrolled in hospice were less likely to have received chemotherapy within 14 days of death compared with those who never enrolled (3.5% vs 13.5%; *P* < 0.0001). Hospice enrollees were also less likely to have an acute care hospitalization within 30 days of death (36.2% vs 71.0%; *P* < 0.0001) and less likely to have at least two hospitalizations within 30 days of death (6.5% vs 18.5%; *P* < 0.0001) compared with those who never had an enrollment. Hospice enrollees were less likely to be admitted to the ICU within 30 days of death (7.4% vs 26.5%; *P* < 0.0001).

We examined predictors of aggressive end‐of‐life care using multivariable logistic regression models (Table 3). In the model for chemotherapy receipt within 14 days of death, patients who were older, female, lived in the South (vs Northeast), or had longer survival were less likely to receive late chemotherapy. Patients were more likely to receive late chemotherapy if they were married, had stage IV disease (vs stage I), died in 2009‐2012 (vs 2000‐2004), or had a higher SES quintile.

**Table 3:** Characteristics associated with end‐of‐life aggressive treatment, 2000‐2012

Characteristic	At least 1 hospitalization within last 30 d (N = 7 479)^b^		ICU admission within last 30 d (N = 2 163)^b^		Chemotherapy within last 14 d (N = 1 056)^b^	
	OR (95% CI)	*P*‐value	OR (95% CI)	*P*‐value	OR (95% CI)	*P*‐value
Age at death (ref = 66‐69)						
70‐74	0.92 (0.83‐1.02)	0.10	0.80 (0.69‐0.92)	0.002	0.86 (0.72‐1.03)	0.09
75‐79	0.81 (0.73‐0.89)	<0.0001	0.76 (0.66‐0.88)	0.0003	0.76 (0.64‐0.92)	0.004
80‐84	0.72 (0.64‐0.80)	<0.0001	0.71 (0.61‐0.83)	<0.0001	0.51 (0.41‐0.64)	<0.0001
85+	0.63 (0.55‐0.71)	<0.0001	0.58 (0.48‐0.70)	<0.0001	0.37 (0.87‐0.50)	<0.0001
Sex (ref = male)						
Female	0.85 (0.80‐0.91)	<0.0001	0.88 (0.80‐0.97)	0.01	0.84 (0.73‐0.96)	0.009
Race/ethnicity (ref = white)						
Black	1.23 (1.09‐1.38)	0.0006	1.14 (0.97‐1.35)	0.12	0.81 (0.63‐1.06)	0.12
Hispanic	1.04 (0.84‐1.30)	0.72	1.10 (0.81‐1.48)	0.55	0.75 (0.45‐1.25)	0.27
Asian/other	1.09 (0.95‐1.26)	0.23	1.16 (0.96‐1.40)	0.13	0.91 (0.68‐1.22)	0.53
Marital status (ref = unmarried)						
Married	1.13 (1.06‐1.23)	0.0004	1.11 (1.004‐1.23)	0.04	1.41 (1.22‐1.63)	<0.0001
Unknown	0.86 (0.71‐1.03)	0.10	1.01 (0.77‐1.33)	0.92	0.82 (0.53‐1.28)	0.38
AJCC stage (ref = I)						
II	1.16 (1.01‐1.33)	0.03	1.20 (0.98‐1.46)	0.08	1.83 (1.23‐2.72)	0.003
III	1.06 (0.90‐1.25)	0.50	0.94 (0.74‐1.21)	0.64	1.48 (0.94‐2.33)	0.10
IV	1.33 (1.16‐1.52)	<0.0001	0.99 (0.81‐1.21)	0.93	2.35 (1.59‐3.47)	<0.0001
Charlson score (ref = 0)						
1	1.13 (1.05‐1.22)	0.002	1.11 (0.99‐1.25)	0.09	1.02 (0.88‐1.19)	0.81
2+	1.43 (1.32‐1.54)	<0.0001	1.62 (1.45‐1.81)	<0.0001	0.91 (0.78‐1.07)	0.24
Year of death (ref = 2000‐2004)						
2005‐2008	1.10 (1.02‐1.19)	0.01	1.41 (1.25‐1.59)	<0.0001	1.10 (0.94‐1.28)	0.23
2009‐2012	1.13 (1.05‐1.23)	0.002	1.79 (1.59‐2.02)	<0.0001	0.92 (0.78‐1.09)	0.34
SEER region (ref = Northeast)						
South	0.84 (0.76‐0.93)	0.0005	0.92 (0.79‐1.07)	0.26	0.81 (0.66‐0.99)	0.04
Midwest	0.84 (0.75‐0.94)	0.002	0.76 (0.64‐0.90)	0.002	0.82 (0.66‐1.02)	0.08
West/Hawaii	0.83 (0.76‐0.90)	<0.0001	1.11 (0.98‐1.25)	0.10	0.87 (0.74‐1.02)	0.10
Residence (ref = large metro)						
Metro/urban	0.81 (0.75‐0.87)	<0.0001	0.62 (0.56‐0.69)	<0.0001	0.92 (0.80‐1.05)	0.22
Less Urban/rural	0.83 (0.73‐0.94)	0.003	0.51 (0.41‐0.63)	<0.0001	0.79 (0.60‐1.03)	0.09
SES (ref = 0)^a^						
1	0.89 (0.80‐0.98)	0.02	0.94 (0.82‐1.09)	0.41	1.13 (0.91‐1.41)	0.26
2	0.89 (0.80‐0.98)	0.02	0.91 (0.78‐1.05)	0.20	1.34 (1.08‐1.66)	0.008
3	0.85 (0.77‐0.94)	0.002	0.89 (0.77‐1.04)	0.14	1.16 (0.93‐1.44)	0.19
4 (highest)	0.84 (0.76‐0.94)	0.002	0.84 (0.72‐0.98)	0.03	1.46 (1.17‐1.81)	0.0006
Pancreas location (ref = head)						
Tail/body	0.94 (0.87‐1.02)	0.14	0.81 (0.72‐0.92)	0.0008	1.11 (0.94‐1.30)	0.22
Other	0.99 (0.91‐1.08)	0.85	0.90 (0.80‐1.02)	0.09	1.20 (1.02‐1.41)	0.03
Survival (mo)	0.98 (0.98‐0.99)	<0.0001	0.99 (0.98‐0.99)	<0.0001	0.98 (0.97‐0.99)	<0.0001

Census tract quintile.

AUC: 0.61 (late hospitalizations), 0.63 (late ICU), 0.65 (late chemotherapy).

For the model analyzing predictors of at least one hospitalization at the end‐of‐life, patients who were Black (vs White), married, had stage IV disease (vs stage I), with a higher Charlson score (vs 0), or died in later years (vs 2000‐2004) were more likely to have a hospitalization, while older patients, females, or those who had longer survival were less likely to be hospitalized within 30 days of death. Similar results were seen for the outcome of late ICU admissions: patients who were married, had a Charlson score ≥1, or died in later years were more likely to be admitted to the ICU within 30 days of death. Patients were less likely to have a late ICU admission if they were older, female, lived in the Midwest (vs (Northeast), lived in a metro/urban or less urban/rural area (vs large metro), were in the highest SES quintile, had a tumor in the pancreas tail/body (vs head), or longer survival (Table 3).

## 4 DISCUSSION

We utilized SEER‐Medicare to study patterns of hospice utilization and aggressive end‐of‐life care among older patients with PDAC. We found that nearly three‐fourths of patients with PDAC receive hospice services prior to death, with an increasing trend from 2000 to 2012. However, over one‐fourth of those who enrolled did so within the last 7 days of life, suggesting that a substantial proportion of patients are receiving hospice services late in the disease process. Importantly, we also demonstrated high rates of aggressive end‐of‐life care, including considerably high proportions of patients with chemotherapy use and hospitalizations in their final days of life. Notably, rates of ICU admissions continued to rise during our study period. Collectively, our results provide valuable evidence supporting the need for efforts to enhance end‐of‐life care for patients with PDAC.

Despite improvements in hospice rates over time, our study suggests that patients still receive these services late in the disease course. Importantly, rates of hospice utilization for patients in our study are higher than those seen in other cancers over a similar period, including acute myeloid leukemia (44.4%),^30^ breast cancer (62.8%),^39^ and hepatocellular carcinoma (63.0%).^40^ Notably, this aligns with prior work demonstrating that among eight different cancer types, pancreatic cancer patients had the highest rates of hospice enrollment.^24^ Clinically, the finding that PDAC patients have higher hospice enrollment may potentially be related to these patients presenting with a high symptom burden and the relatively limited treatment options for this population. Future research should seek to determine the optimal timing of hospice for patients with PDAC. We found a substantial proportion of patients are receiving hospice services within their final week of life, when the benefits of hospice may be more limited. Ultimately, our results demonstrate the need for additional investigations to help better understand potential barriers to hospice enrollment and determine the most appropriate end‐of‐life services for these patients.

Importantly, we investigated rates of aggressive end‐of‐life treatment and found that nearly half of patients with PDAC experienced at least one hospitalization in the final month of life, with one‐tenth experiencing two or more hospitalizations during this time. This finding is critically important, as studies have shown that patients with cancer wish to avoid hospitalizations at the end‐of‐life,^41^, ^42^, ^43^, ^44^, ^45^ and additional research is needed to help better align care with patient preferences. In addition, we found that over 10% of patients had a late ICU admission, and the proportion of patients experiencing this outcome increased over time. Clinically, PDAC patients often experience high rates of infectious complications as well as biliary and bowel obstructions, which can result in acute clinical deterioration, and may help explain high rates of ICU admissions in this population.^46^, ^47^ Our finding that the use of ICU care has been increasing for this population provides important evidence supporting the need for interventions to help reverse this trend. Overall, receipt of aggressive care appeared to remain stable to worse over our study period, and our findings highlight areas for future interventions targeting ways to optimize care at the end‐of‐life for this population.

Interestingly, we identified predictors of hospice use and aggressive end‐of‐life care among patients with PDAC. We found that female patients were more likely to enroll in hospice, less likely to have a late enrollment, and less likely to receive aggressive end‐of‐life treatment compared with male patients. This is hypothesis‐generating, but research has suggested that female patients may be more likely to seek out and receive supportive care services compared with males.^48^, ^49^, ^50^ We also demonstrated that Black and Asian patients were less likely to enroll in hospice compared with White patients. These variations in hospice enrollment may indicate a difference in how patients and clinicians consider and make treatment decisions, and additional research is needed to further investigate patient‐clinician discussions about end‐of‐life care preferences and decisions.^51^, ^52^, ^53^ Notably, our findings are consistent with prior studies of end‐of‐life care disparities, both in patients with PDAC as well as other cancer types, which have demonstrated that minority patients were less likely to enroll in hospice.^26^, ^38^, ^54^

Previous studies have shown differences in hospice enrollment and end‐of‐life treatment based on geographic location.^55^, ^56^, ^57^ Our analysis also suggests that regional location plays a role in hospice enrollment and treatment at end‐of‐life. Hospice enrollment was higher among patients in the Midwest and South (compared to Northeast). In contrast, patients in the Midwest were less likely to have a late ICU admission and patients in the South were less likely to have late chemotherapy receipt. Our study highlights the need for further study on the mechanism behind these regional differences among patients with PDAC.

Our study has several limitations. The SEER‐Medicare database only includes patients with Medicare coverage; thus, our results may not be generalizable to the entire PDAC population. However, pancreatic cancer is more commonly diagnosed at older ages, with the majority of patients aged 65 or older.^2^ This analysis also only includes follow‐up to 2012 and may not reflect current practice or newer treatments. We also do not include every indicator of aggressive end‐of‐life care. Future work should include investigations on other possible indicators, such as emergency room visits and hospital length of stay. Medicare does not provide information on whether treatment was prescribed for curative or palliative intent, and we lack data about patient preferences and patient‐clinician discussions about end‐of‐life care. Therefore, we cannot comment on the appropriateness of treatment at the individual level. Future research should seek to prospectively investigate associations between patient preferences for care, patient‐clinician discussions about care preferences, and receipt of hospice services and aggressive care at the end‐of‐life for patients with PDAC.

In conclusion, we found that hospice use among patients with PDAC is high and continues to increase, but a substantial proportion are still not receiving hospice services, or the services are only provided in the final week of life. Additionally, ICU admissions and multiple hospitalizations within the final 30 days of life continued to show an increase over time. We also highlighted patient sociodemographic and clinical characteristics associated with the use of hospice services and the receipt of aggressive end‐of‐life care, which can inform future studies to provide personalized care tailored to the needs of these subgroups. Collectively, our findings underscore the need for additional work to improve end‐of‐life care among these patients and we provide important information to inform future research efforts targeting patients at risk for suboptimal end‐of‐life care.

## CONFLICT OF INTEREST

None declared.

## AUTHOR CONTRIBUTIONS

ACT, RN, CYK, CH: conceptualization, formal analysis, writing—original draft, and writing—review and editing.
