# Supplementary figures and images for: Patterns and predictors of end‐of‐life care in older patients with pancreatic cancer
Source: Cancer Med. 2018 Nov 13;7(12):6401–10. doi: 10.1002/cam4.1861 (PMC6308041; doi:10.1002/cam4.1861)

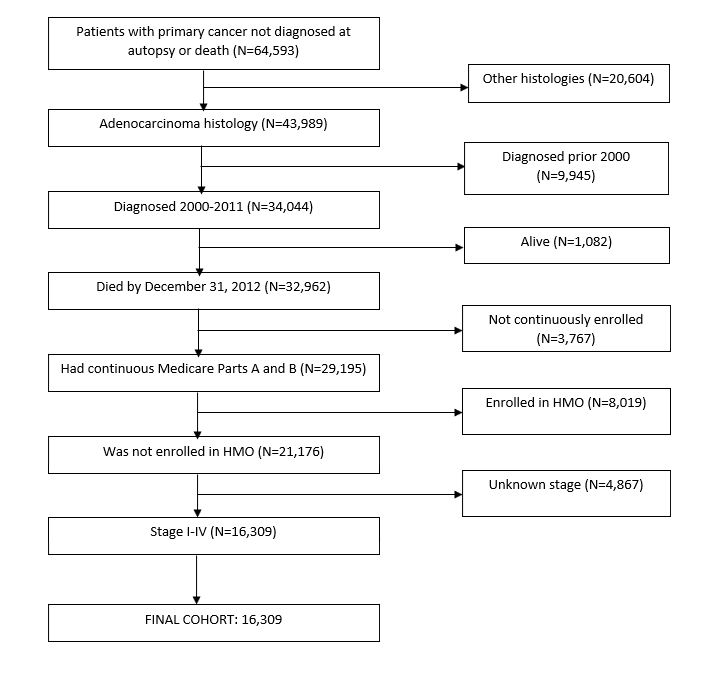

Supplement: Supplementary file 1 [file CAM4-7-6401-s001.tif]
