# Supplementary material for: Patterns and predictors of end‐of‐life care in older patients with pancreatic cancer
Source: Cancer Med. 2018 Nov 13;7(12):6401–10. doi: 10.1002/cam4.1861 (PMC6308041; doi:10.1002/cam4.1861)
Supplement: Supplementary file 2 [file CAM4-7-6401-s002.docx]

**Appendix**

**Database description:**

The SEER database includes patient-level information on cancer patients from specific geographic regions that represent approximately 28% of the U.S. population.^1^ This database includes information on a patient’s demographics, cancer site, histology, stage, diagnosis date, first course of treatment, and date of death. The Medicare database includes claims data for inpatient, outpatient, physician, and hospice services for approximately 97% of Medicare enrollees.^2^ The SEER-Medicare database links these two databases for patients 65 and older and can be used to examine patterns of care over time.^2^

1. Howlader N, Noone AM, Krapcho M, et al. SEER Cancer Statistics Review, 1975-2014, National Cancer Institute. Bethesda, MD, <https://seer.cancer.gov/csr/1975_2014/>, based on November 2016 SEER data submission, posted to the SEER web site, April 2017.

2. Warren JL, Klabunde CN, Schrag D, Bach PB, Riley GF. Overview of the SEER-Medicare data: content, research applications, and generalizability to the United States elderly population. Med Care. 2002;40: IV-3-18.

**Appendix Figure 1: Cohort Inclusion/Exclusion Criteria**

**Appendix Table 1. Care Definitions, Medicare Billing and SEER Codes**

| **Variable** | **Definition** | **Codes** |
| --- | --- | --- |
| Histology | ICD-O-3 code in SEER | ICD-O-3: 8050, 8140-8147, 8160-8162, 8180-8221, 8250-8507, 8514, 8520-8551, 8560, 8570-8574, 8576, 8940-8941 |
| Radiation therapy | a. At least one Medicare claim between diagnosis and death  b. At least one claim within 14 days of death | ICD-9-CM: V58.0, 92.21-92.29  CPT: 7331-7336, 73399, 77400- 77499, 77750- 77799  Revenue Center: 0330, 0333, 0339 |
| Chemotherapy | a. At least one Medicare claim between diagnosis and death  b. At least one claim within 14 days of death | ICD-9-CM: V58.1, 99.25  HCPCS: C1166, C1168, C1179, C9110, C9205, C9207, C9213-C9216, C9411, C9414-C9419, C942x, C9430-C9438, G0345-G0363, J9000–J9999, Q0083–Q0085  CPT: 9651x-9654x, 964xx  Revenue Center 0331, 0332, 0335 |
| Hospitalization | a. At least one acute care hospitalization inpatient claim between diagnosis and death  b. At least one acute care hospitalization inpatient claim within 30 days of death | N/A |
| ICU admission | a. At least one ICU inpatient claim between diagnosis and death  b. At least one ICU inpatient claim within 30 days of death | Intensive care revenue center code 020x |
| Hospice | a. At least one hospice claim between diagnosis and death  b. At least one hospice claim within 7 days of death | N/A |

**Appendix Table 2: Characteristics Associated with Hospice Enrollment Within 3 Days of Death***

|  | **Hospice Enrollment, 3 days (N=1735)** | |
| --- | --- | --- |
| **Characteristic** | **OR (95% CI)** | **p-value** |
| Age at death (ref=66-69) |  |  |
| 70-74 | 0.70 (0.60-0.83) | <0.0001 |
| 75-79 | 0.72 (0.62-0.84) | <0.0001 |
| 80-84 | 0.60 (0.51-0.72) | <0.0001 |
| 85+ | 0.51 (0.41-0.62) | <0.0001 |
|  |  |  |
| Sex (ref=Male) |  |  |
| Female | 0.77 (0.69-0.86) | <0.0001 |
|  |  |  |
| Race/Ethnicity (ref=White) |  |  |
| Black | 1.01 (0.83-1.23) | 0.93 |
| Hispanic | 0.84 (0.56-1.27) | 0.40 |
| Asian/Other | 1.09 (0.85-1.40) | 0.52 |
|  |  |  |
| Marital Status (ref=Unmarried) |  |  |
| Married | 1.17 (1.04-1.31) | 0.009 |
| Unknown | 1.06 (0.79-1.44) | 0.69 |
|  |  |  |
| AJCC Stage (ref=I) |  |  |
| II | 0.92 (0.73-1.16) | 0.47 |
| III | 0.83 (0.62-1.10) | 0.19 |
| IV | 1.14 (0.91-1.43) | 0.27 |
|  |  |  |
| Charlson Score (ref=0) |  |  |
| 1 | 1.07 (0.94-1.21) | 0.29 |
| 2+ | 1.16 (1.03-1.32) | 0.02 |
|  |  |  |
| Year of Death (ref=2000-2004) |  |  |
| 2005-2008 | 1.02 (0.89-1.16) | 0.81 |
| 2009-2012 | 1.13 (0.99-1.29) | 0.06 |
|  |  |  |
| SEER Region (ref=Northeast) |  |  |
| South | 0.67 (0.57-0.79) | <0.0001 |
| Midwest | 0.87 (0.73-1.03) | 0.10 |
| West/Hawaii | 0.70 (0.61-0.80) | <0.0001 |
|  |  |  |
| Residence (ref=Large Metro) |  |  |
| Metro/Urban | 0.92 (0.82-1.03) | 0.14 |
| Less Urban/Rural | 0.65 (0.52-0.81) | <0.0001 |
|  |  |  |
| SES** (ref=0) |  |  |
| 1 | 0.995 (0.97-1.27) | 0.95 |
| 2 | 0.94 (0.84-1.18) | 0.51 |
| 3 | 0.95 (0.79-1.12) | 0.57 |
| 4 (highest) | 1.01 (0.80-1.13) | 0.93 |
|  |  |  |
| Pancreas Location (ref=Head) |  |  |
| Tail/Body | 0.96 (0.84-1.10) | 0.53 |
| Other | 1.11 (0.97-1.27) | 0.13 |
|  |  |  |
| Survival (months) | 0.99 (0.98-0.998) | 0.02 |

*Models significant after Bonferroni correction

******Census tract quintile

***AUC: 0.60
